# A Manual of Biological Therapeutics

**Published:** 1915-02

**Authors:** Malone Duggan


A MANUAL OF BIOLOGICAL THERAPEUTICS. Parke, Davis & Co., 1914.

This little manual presents in a very comprehensive and concise style the elementary essentials as now taught on the subjects of sera, bacterins, phylacogens, tuberculins, glandular extracts, toxins, cultures, antigens, etc. It may be rightfully regarded as the A, B, C's of this modern therapy. As such, it has a distinct mission. To many successful and busy practitioners these subjects are but theories, and not practically understood. This volume is certain to stimulate interest in this important field, and in doing so will prove a real gain to medicine. MALONE DUGGAN.

